# Seeking to improve criterion and construct validity of retrospective self-reports of physical activity with brief reporting periods and quantitative response scales

**DOI:** 10.1038/s41598-025-16741-8

**Published:** 2025-09-29

**Authors:** Arthur A. Stone, Meynard Toledo, Stefan Schneider, Joshua Smyth, Doerte U. Junghaenel, Sarah Goldstein, Olivia Pomeroy

**Affiliations:** 1 Dornsife Center for Self-Report Science, University of Southern California, Los Angeles, USA; 2 Department of Psychology, University of Southern California, Los Angeles, USA; 3 Ohio State University, Columbus, USA

**Keywords:** EMA, ESM, Criterion validity, Construct validity, Self-report, Accelerometry, Physical activity, Sedentary behavior, Between-persons analyses, Psychology, Human behaviour

## Abstract

Self-reported information is central to many scientific fields and achieving high levels of validity for these data is paramount. Concerns have been voiced about the validity of retrospective self-reports because of possible distortion of recall by memory failures and cognitive heuristics. In this study we test two techniques with the potential to improve self-report validity: the use of brief reporting periods (near-momentary reports) and of quantitative response scales (duration in minutes). To evaluate validity, a set of self-reported physical activity outcomes were compared with objectively assessed physical activities. A total of 258 community-dwelling adults self-reported their momentary physical activity 5 times per day and wore accelerometers throughout 7 days; they also provided end-of-week assessments of physical activity. Regarding criterion validity, all self-reports indicated lower duration of sedentary behavior, however, momentary reports were closer in magnitude to accelerometry than were 1-week recall reports. All self-reports indicated greater duration of physical activity than accelerometry. Correlations between self-reports and accelerometry data were low to modest for momentary and retrospective reports; they were also low for reporting with quantitative versus relativistic, Verbal Response Scales, with the exception of momentary reports of sedentary duration (*r* = .61). Regarding construct validity, associations between most demographic variables and self-reports did not replicate those observed for accelerometry-based measures; in some cases, these associations showed opposite directions, indicating poor validity. In sum, apart from two criterion validity analyses showing higher validity for momentary reporting (compared with recall), the results did not support the hypotheses that the use of brief reporting periods and quantitative response scales improved validity over 7-day reporting and Verbal Response Scales, respectively. Particularly concerning was the poor performance of all methods in tests of construct validity.

## Introduction

Human subject research regularly incorporates self-reports from individuals studied in many scientific fields including healthcare, behavioral science, epidemiology, and political polling, to name just a few. Self-reporting is a cost-effective and convenient way of obtaining all manner of information about people, such as their demographics, symptoms, living conditions, stressors, health habits, social interactions, political opinions, working conditions, and so forth. Self-reports, though, can be susceptible to error and bias, and many reports have acknowledged poor to moderate validity for retrospective self-reports (that is, reporting that covers a substantial period, such as a week, a month, or longer) when objective reference standards are available for comparison^1–5^. For these reasons, behavioral scientists and survey experts conduct research to improve self-reporting.

This report examines the validity of self-reports that are meant to characterize the behavior and experiences of individuals over a defined period. Examples of such reports are level of depressed affect, the frequency of their cigarette consumption, and the amount of pain experienced over the course of a week. These data afford between-person analyses, for instance, group comparisons by demographic variables or relevant risk factors. Between-person analyses are conceptually distinct from analyses of repeated measures from individuals which enable investigation of within-person associations; further, between- and within-persons analyses are conceptually independent. Person-level behavioral and experiential data are usually conceptualized as including phenomena that occur during the waking hours for the period considered: how much pain was experienced during the waking hours of the last 7 days is a good example. (There can be exceptions, such as assessments that focus on nighttime occurrences.)

To facilitate validity analyses, it is very useful to have an objectively measured reference variable for comparison with self-reports. The domains we chose to study are sedentary behavior (SB) and physical activity (PA). We operationally defined SB as any waking behavior performed in a sitting, reclining, or lying posture, as classified by accelerometry. Moreover, we operationally defined PA as any bodily movement produced by skeletal muscles that result in energy expenditure, which are detectable by accelerometers. This typically includes activities such as walking, running, and other forms of ambulatory movement^6–8^.. Although we acknowledge that accelerometers only quantify body acceleration rather than capture all forms of activities that result in energy expenditure, they provide generally well-accepted and widely used metrics in the field of physical activity research for objectively assessing PA and SB behaviors. By obtaining self-reports of comparable SB and PA with retrospective and momentary self-reporting and with accelerometry, we could contrast the self-reports with objective measures for the same period, enabling us to advance strong statements about validity. It is notable that such comparisons are not possible for many self-reported measures, because there are no established, objective reports of self-reported states (e.g., affect, pain, and symptoms).

The most typical method of measuring self-reported SB and PA is with recall measures wherein individuals are asked to report the intensity and/or duration of SB and PA based on their memories from a past period (for a review, see Sylvia, et al.^9^,Doma, et al.^10^). Studies of retrospective self-reporting of SB and PA with reporting periods of 7 days show that they do not replicate objective reports from accelerometry. First, the magnitude of direct associations between accelerometry and 7-day retrospective recall are weak to moderate (correlations in the range of 0.1 to 0.4). Second, associations of self-reports with demographic variables, such as age, gender, and education, often differ from those observed with objective measurements, which we interpret as challenging the construct validity of retrospective self-reports.

To take one example, Dyrstad, et al.^11^ studied 1,751 adults in Norway who wore accelerometers for one week and retrospectively self-reported their SB and PA with a standardized, physical activity questionnaire. For associations with age, objective counts of steps per day (a standard measure of physical activity) increased over the first three age categories (8049, 8394, and 8491 steps/day), but in the last category (65 + years) step counts were many fewer (7242 steps/day). In contrast, self-reported total activity duration followed a strikingly different pattern: it was similar for the first two age categories (69 and 63 min/day), increased in the third category (78 min), and was highest in the fourth category (84 min). Sedentary behavior (sedentary minutes from accelerometry and sitting minutes from self-reports) also showed an opposite pattern. Although not all studies show these puzzling patterns, the fact that they are present in some studies is cause for concern.

Given the desire for increased validity in self-reports of SB and PA, we tested methods for improving the validity of self-reporting for comparisons between individuals and two features of self-reporting techniques with potential to do so were evaluated. The first technique reduced the length of recall used in questions (the reporting period) with the intent of mitigating biases associated with cognitive heuristics and error connected with extended reporting periods. This widely used momentary data acquisition technique is known as Ecological Momentary Assessment (EMA)^1–5^ and the Experience Sampling Method^5,12,13^. Instead of asking about activity for the previous 7 days or longer as in recall assessment, the momentary approach employed here asked about the last 5–120 min, significantly decreasing the reporting interval. Momentary questions were administered many times over the course of a week as respondents carried out their everyday activities. Responses were summarized to create a person-level value representing physical activity over the waking days of the week. The recall approach were assessments of activity over the same 7-day period. Thus, the first factor was momentary versus recall reporting.

The second technique for improving self-report examined the response scales for the construct being assessed. We examined the self-reported *intensity* of PA and SB with Verbal Response Scales (VRS) and the self-reported *duration* of PA and SB with quantitative scales asking about minutes of engagement in SB and PA. Prior work on VRSs indicated that they are susceptible to bias because response options for these scales are relativistic (Schwarz^14^, but also see Walentynowicz, et al.^15^ and Schneider and Stone^16^). For instance, a common VRS scale set of response options is “None,” “Slightly,” “Moderately,” and “Extremely,” though the number of labels and the descriptors may vary according to the application. These descriptors refer to relative magnitude of the phenomenon in question as opposed to describing an absolute level of magnitude (relativistic as in “A *very large* mouse is much smaller than a *very small* elephant” given the average size of mice and elephants). An alternative technique for assessing magnitude are quantitative scales that yield ratio level data, such as currency, length, and duration. These scales are not relativistic, because if used properly, they correspond to the absolute amount of the thing being measured. In this study PA and SB were measured with both VRS and quantitative duration scales, allowing direct tests of the concordance of VRS versus a duration response scale in comparison to the accelerometry reference standard.

We advance a series of hypotheses based on these two techniques. The first two hypotheses address concurrent criterion validity^17^, examining the association of a new measure with a known reference standard for the construct of interest. The first hypothesis (H1) addressed the effect of decreasing the duration of the reporting period to momentary assessment from 1-week recall. We hypothesized that (H1a) aggregated momentary reports for a week are more accurate on average, that is, that the levels of momentary SB and PA are closer to the accelerometry levels than are the 1-week recall levels, on average across respondents. These analyses were only possible for self-reports using quantitative response scales, because the self-reports with VRSs are not comparable with accelerometry-derived values, rendering mean level differences uninterpretable. We also hypothesized that (H1b) aggregated momentary reports for a week are more strongly associated with accelerometry compared to the association between 1-week recall reports and accelerometry.

The second criterion validity hypothesis (H2) tested whether the quantitative scales were more strongly associated with accelerometry relative to the strength of the associations with VRSs. We compared correlations between accelerometry measures of SB (or PA) and quantitative (minutes) versus VRS scales. We hypothesized that criterion validity would be higher for the quantitative scales relative to the VRS scales.

The last set of hypotheses (H3) addressed an aspect of construct validity^18^ and tested the associations between each of the self-report variables with demographic characteristics of the sample, such as age and gender. The comparison we focus on is how these associations compare with the “true” associations, namely, between accelerometry and the same demographic variables. Regarding the reporting periods (H3a), we hypothesized that EMA self-reports with brief recall periods would generate associations more like those from accelerometry than the associations with 7-day recall. Regarding the VRS versus quantitative response scales (H3b), we hypothesized that the quantitative scale would generate associations closer to those from accelerometer versus VRS. In the best-case scenario for supporting the construct validity of self-reports, we expect that PA and SB self-reports reproduce the magnitudes and directionalities of the accelerometry associations; in other words, that accelerometry and self-reports relate in very similar ways to external criteria. If this was not the case, then self-reports might not be suitable substitutions for accelerometry. The strength of any conclusions depends upon the degree of non-invariance between the measures. The idea is similar to the concept of predictive invariance^19^, where comparable measures should have identical or very similar associations with criterion variables.

For H3, the variables correlated with physical activity were five standard demographic questions: age, income, education, gender, and race. As previously mentioned, epidemiological studies have consistently demonstrated significant variations in physical activity levels across strata of these demographic characteristics^6,20,21^. Although there may be reporting error in answers to these demographic questions, the data is generally viewed as sound, unless there are very strong demand characteristics in play encouraging respondents to distort their responses.

## Materials and methods

All study procedures were reviewed and approved by the Biomedical Research Alliance of New York Institutional Review Board (#22-183-1044) and all research was performed in accordance with relevant guidelines and regulations. Each participant provided an electronic informed consent prior to participation.

Participants. This study utilized data from an EMA study investigating the relationship between self-reported and accelerometry-measured physical activity behaviors. Participants were drawn from the Understanding America Study (UAS^22^;), a nationally representative internet panel comprising approximately 13,000 U.S. residents. The UAS employs address-based sampling to recruit participants, ensuring representation from diverse socioeconomic backgrounds. Notably, individuals lacking internet access are provided with tablets and broadband connectivity to promote inclusivity. This recruitment strategy mitigates the limitations of traditional online panels, which often over-represent certain demographics. Individuals from the UAS panel were eligible for this EMA study if they met the following inclusion criteria: (1) were fluent in English; (2) were 18 years of age or older; (3) had no hearing impairments, (4) had no vision impairments that could not be corrected with contact lenses or glasses; (5) did not work a night shift; (6) were not on bed-rest; and (7) did not require any mobility devices to be able to move around. Potential participants were initially contacted through email and interested participants were asked to complete a screening survey to assess their eligibility for the study.

Of the 1,363 individuals contacted, 1,021 met the inclusion criteria. Following the screening process, 407 individuals provided informed consent and were enrolled in the study. A total of 359 participants completed the study protocol; however, only 258 had complete accelerometer, EMA, and 7-day recall data. The final analytic sample was 49.2% female, averaged 48.6 (14.7) years of age, was 79.5 white and 6.6% black, 58.1% achieved a bachelor’s degree or higher, and 45.3% had a household income of at least $100,000. There were no statistically significant differences in these key demographic characteristics between participants included in the final analytic sample (*N* = 258) and those who were excluded due to incomplete data (*N* = 101). (A similar reduction of sample size has been seen in at least one prior self-report with accelerometry study^11^).

Study procedures. The study employed a fully remote 7-day EMA protocol. Consented participants received a package containing a smartphone, charger, activity monitor, and materials (including instructions) for returning the devices upon study completion. A day prior to the start of their 7-day protocol, participants were required to complete a baseline questionnaire and watch a 20-minute instructional video detailing the proper use of the activity monitor and the EMA survey application. Participants who failed to complete this activity were asked to return their study package and reschedule their participation start date. Over the following 7 days, participants received 5 EMA prompts per day. Participants were randomly assigned to either of the two study conditions where they were asked to report on their activities either “during the 5 minutes before the prompt” or “during the 2 hours before the prompt.” To ensure compliance and address technical issues, research staff monitored EMA responses daily and provided support via telephone and email. Participants also received daily email reminders to complete study activities. Simultaneously, they were also asked to continuously wear the activity monitor. At the end of the 7-day period, participants completed a final survey which asked them to self-report their physical activity behaviors over the entire 7-day period. Upon completion, participants returned the study materials using a provided prepaid shipping label. Compensation of up to $200 was provided, prorated based on the level of protocol completion.

Measures. Participants provided demographic information, including age, education level, gender, race, marital status, and income in the baseline questionnaire. These variables were considered outcomes in the analyses. Physical activity levels were assessed on the final survey using a researcher-developed physical activity questionnaire that asked participants to recall and report the total time (in minutes) they spent in five activity categories (i.e., sedentary, standing, light physical activity, moderate physical activity, and vigorous physical activity) over the last 7 days. To quantify total physical activity behaviors, we calculated the sum of the reported minutes from the light, moderate, and vigorous activity categories. Participants also rated their overall level of sedentariness and physical activity over the same 7-day period using a 5-point verbal scale (not at all – extremely).

EMA questions were administered via the movisensXS app on dedicated smartphones (Motorola G Power 2021). Participants received five EMA prompts per day, delivered at random times between 6:00 AM and 10:00 PM in their local time zone. To ensure adequate spacing, prompts were scheduled at least 15 min apart. Five EMA items asked participants to report the number of minutes spent (1) sitting or lying down (i.e., sedentary), (2) standing, (3) in LPA, (4) in MPA, and (5) in VPA during the targeted time period. These items were designed to align with the activity variables captured by the activity monitor. Participants responded using an open-ended numeric format. Two additional items asked participants to rate their overall perceived sedentariness and physical activity during the targeted reporting period using a 5-point verbal scale (not at all – extremely). At the first EMA prompt each day, participants also reported their sleep and wake times. Finally, the EMA questionnaire also included items on affect, pain, and fatigue, but these were not analyzed in the current study. Participants had a 15-minute window to respond to each EMA prompt. After an initial 1-minute alarm, reminder alarms were issued at 5 and 10 min. If a participant failed to respond within 15 min, the prompt expired, and the survey could not be completed. To ensure reliable weekly estimates of PA and SB from the EMA prompts, only participants who responded to at least 50% (17 out of 35) of the scheduled EMA prompts were included in the final analytic sample. The overall EMA compliance rate, defined as the total number of valid EMA responses over the number of prompts received during their wake period, for the analytic sample was 93.6%.

To objectively measure physical activity, participants wore an activPAL™ accelerometer on the anterior midline of their thigh continuously for seven days, except during water-based activities. Unlike traditional accelerometers worn on the hip or wrist, the activPAL provides accurate data on posture- (e.g., sitting/standing) and movement-based (e.g., stepping) behaviors e.g.^23,24^,. Sleep intervals were estimated using a weighted average algorithm^25^ that considered information from both self-reported (EMA-derived) and activPAL-determined sleep and wake times. Briefly, the algorithm combined measurements of awakening (and bedtime) from activPAL and self-reported sleep times from the EMA; convergence on a value was taken as relatively strong evidence for the true underlying sleep/wake time. When they did not converge, the two estimates were weighted according to their likelihood based on the empirical distribution of all measurements of the sleep/wake time across the sleep history of the individual. All days when there was at least a two-hour difference between the activPAL-determined and EMA-reported sleep/wake times were manually examined by two independent members of the research team, and a consensus time was determined. All periods identified as sleep were excluded from the analysis. In addition, periods of non-wear identified by ActivPAL software (PALbatch v8.10.12.60, PAL Technologies Ltd, Scotland, UK) were excluded from the analyses. The activPAL classifies activity into sedentary behavior (sitting or lying down); standing (upright posture without ambulation); and stepping (ambulation, including walking and running). Using step cadence (steps per minute), stepping activity was further categorized into light physical activity (LPA; cadence < 100 steps/minute), moderate physical activity (MPA; cadence 100–125 steps/minute), and vigorous physical activity (VPA; cadence > 125 steps/minute)^26^. The total time spent in sedentary and active behaviors (as sum of the light, moderate, and vigorous activities) over the 7-day period were calculated for each participant.

It is important to acknowledge that although the activPAL is highly effective for classifying these postural and ambulatory behaviors, its primary mechanism relies on detecting movement and changes in thigh position; consequently, like other accelerometers, it may not fully capture physical activities involving significant muscular effort with minimal bodily displacement, such as static isometric exercises or certain types of stationary resistance training. However, for the primary aims of this study—which involve examining patterns of daily sedentary behavior and ambulatory physical activity to compare with self-report methods—the activPAL’s established accuracy in these specific Domains, coupled with the fact, mentioned earlier, that such accelerometer-based devices are widely used and well-accepted objective measures in the field, makes it a suitable objective criterion. On average participants wore the device for 99± 3% of their waketime period; non-wear periods were imputed so as to preserve the relative ratio between activity categories observed on the wear periods.

*Analysis.* Basic demographic characteristics are first presented. To estimate each participant’s overall physical activity levels from the EMA data, we first calculated the total time they reported spending in sedentary and active behaviors across all their EMA responses. Then, we extrapolated this to a weekly estimate by adjusting according to each participant’s total wake time for the week. Specifically, we multiplied the total time spent in each behavior with the ratio of their total weekly wake time to the total time covered by their EMA responses (total number of EMA prompts multiplied by the duration of each EMA reporting period). For the EMA VRS questions (for PA and SB), we took the average ratings across all EMA responses.

To address the concurrent criterion validity hypothesis concerning length of reporting period, H1a, differences between Sedentary Minutes for Recall, EMA, and Accelerometry were tested for significance with paired t-tests for differences between the self-report levels and the accelerometry levels. Paired t-tests were also used to compare level differences between Recall and EMA. To address the criterion validity hypothesis concerning length of reporting period based on associations, H1b, correlations were computed for the Recall and EMA reports and accelerometry measures of the same constructs, and differences in their magnitudes were tested using z-tests for comparing dependent correlation coefficients after Steiger^27^.

To test the criterion validity hypothesis about associations for the response formats (H2), we compared the correlation between accelerometry and self-reports using a quantitative response format (minutes) with the correlation between accelerometry and self-reports using a verbal rating scale (VRS) for measures of the same constructs. Dependent correlation tests were used to test differences in the correlations.

To address construct validity, H3, accelerometry-based, weekly recall-based, and EMA-based physical activity variables (SB and PA) were correlated with each of the demographic variables. This was done separately for momentary versus recall reporting periods and for the quantitative response variables versus for the VRS variables resulting in a total of four comparisons for each demographic. To test H3a, correlation coefficients from the two self-report methods (Recall and EMA) were compared to the coefficients from the accelerometry. To test H3b, correlations coefficients from the two response options (VRS and quantitative) were compared to the accelerometry coefficient. To determine if the two coefficients were statistically different, MANOVAs were computed for each of the outcome variables wherein accelerometry and the four self-reports were outcomes and each demographic variables was a predictor. *Post hoc* tests allowed for evaluation of differences between accelerometry and self-report predictors of outcomes. In addition to conducting individual tests for each demographic variable, we estimated the Mean Absolute Difference (MAD) of the Fisher z-transformed correlations between accelerometry and all demographic variables versus self-report and all demographic variables. The MAD values and their standard errors were derived from the full correlation matrix, as all variables were measured in the same sample. We then conducted z-tests employing the delta method to compare these MAD values between recall and EMA, and between VRS and quantitative responses.

To simplify the analyses and presentation, all categorical demographic variables were dichotomized as follows (age was left a continuous variable): Gender, Male (0), Female (1); Education, Less than a BA (0), BA or greater (1); Income, household income less than $100,000(0), greater than $100,000 (1); and Race, Non-white (0), White (1).

## Results

### Criterion validity of recall period factor (H1)

*H1a.* Levels of SB and PA for self-reports and accelerometry are shown in Table 1. Accelerometry indicated that an average of 4,402 min were spent in SB during week, whereas the means for Recall and EMA minutes were 3,085 and 3,867, which were both significantly lower than the accelerometry mean. (Significant statistical test results are shown in the table and in the table notes.) Moreover, the Recall mean was significantly lower than the EMA mean, whereas the EMA mean was closer in magnitude to the accelerometry mean. The pattern was reversed for PA wherein the accelerometry PA minutes mean was significantly lower, 649, than both Recall PA, 1,656, and EMA PA minutes, 2,453. Furthermore, the Recall mean minutes was significantly lower than the EMA mean, but in this case the Recall mean was closer in magnitude to that of accelerometry.

**Table 1:** Criterion validity: levels of and correlations between Self-report variables and accelerometry measures.

	Mean (SD)	Correlation between Self-report and Accelerometry
Sedentary self-report variables		
Recall sedentary minutes	3,085 (1,596) ^a, *^	.356^e^
EMA total sedentary minutes	3,867 (1,033)^b, *^	.616^e^
Recall average sedentary VRS rating	2.53 (0.91)	0.390
EMA average sedentary VRS rating	2.08 (0.64)	0.381
Total accelerometer sedentary minutes	4,402 (793) ^a, b^	--
Active self-report variables		
Recall active minutes	1,656 (1,223)^c^	0.382
EMA total active minutes	2,453 (1,376)^d^	0.307
Recall average active VRS rating	1.99 (0.81)	0.410
EMA average active VRS rating	1.16 (0.48)	0.325
Total accelerometer active minutes	649 (283)^c, d^	--

All correlations are significant at the *p* < .0001 level.

^a^ indicates that Recall Sedentary Minutes was significantly different from Total Accelerometry Sedentary Minutes at the *p* < .001 level, t(257) = 14.0.

^b^ indicates that EMA Total Sedentary Minutes was significantly different from Total Accelerometry Sedentary Minutes at the *p* < .001 level. t(257) = 10.4.

^c^ indicates that Recall Active Minutes was significantly different from Total Accelerometry Active Minutes at the *p* < .001 level, t(257) = 36.9.

^d^ indicates that EMA Total Active Minutes was significantly different from Total Accelerometry Active Minutes at the *p* < .001 level, t(257)=-22.0.

^e^ indicates that Recall Sedentary Minutes correlation with Total Accelerometry Sedentary was significantly different from the EMA Total Sedentary Minutes correlation with Total Accelerometry Sedentary at the *p* < .001 level, z(257)=-4.81.

^*^ indicates that Recall Sedentary Minutes was significantly different from EMA Total Sedentary Minutes at the *p* < .001 level, t(257) = 8.6.

^#^ indicates that Recall Active Minutes was significantly different from EMA Total Active Minutes at the *p* < .001 level, t(257) = 9.4.

*H1b.* The second tests of criterion validity were correlations computed between self-reported PA and SB with their accelerometry counterparts; correlations are shown in Table 1. For SB, the correlations with Recall sedentary minutes and with EMA sedentary minutes were, respectively, 0.36 and 0.61, each of which is significant. These correlations were significantly different from one another. The pattern and magnitude of correlations for PA were compared with those for SB, 0.38 for Recall minutes and 0.31 for EMA minutes; again, each correlation was significant, but the two were not significantly different (z(257)=-1.24, *p* > .10).

### Criterion validity of response scale factor (H2)

Correlational analyses addressing criterion validity were computed and are presented in Table 1. For SB, there was little difference between the Recall average VRS and the average EMA VRS with correlations of 0.39 and 0.38, respectively (z(257) = 0.17, *p* > .40). However, it is notable that the mean EMA SB minutes was significantly higher than that the recall SB minutes, 0.62 versus 0.36. Turning to PA, the only associations that were different was that the Recall average VRS was marginally higher, 0.41, than the average EMA VRS, 0.31 (z(257) = 1.36, *p* = .087).

### Construct validity for the reporting period and response scale factors (H3)

**Figure 1:**
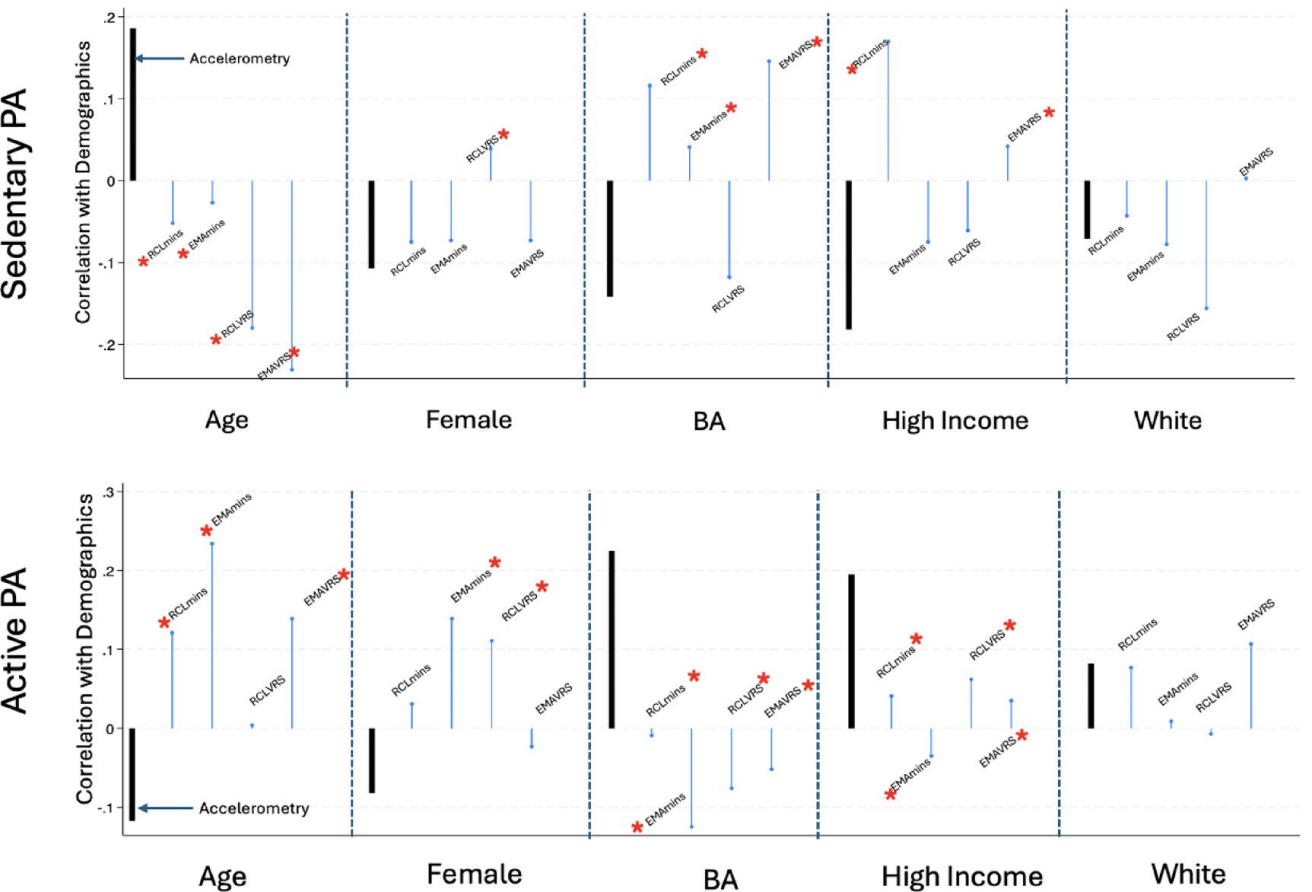
Construct validity: Correlations of recall, EMA, and accelerometry measures with demographic variables

As per the analytic plan, MANOVAs were run for each demographic variable and the results are shown in Table 2. To aid in visualizing the magnitude and direction of the associations, Fig. 1 depicts the associations for each of the five demographic variables and within each section of the figure the correlations between a demographic variable and accelerometry, recall minutes, momentary minutes, recall VRS, and momentary VRS are presented. A red star next to a self-report variable’s label indicates that the correlation between that variable and the criterion was significantly different level from the association between accelerometry and the criterion variable. The top panel shows the sedentary behavior and the lower panel shows physically active behavior.

**Table 2:** Construct validity for all Self-report variables. Correlations of accelerometry and Self-reports of SB and PA with demographic variables.

		Demographic variables				
		Age	Gender: Female	Bachelor’s degree	High income	Race: White
Sedentary behavior	***Accelerometry***	0.186**	***− 0.107***	− 0.142*	− 0.182**	***− 0.071***
	Recall Minutes	− 0.052 #	− 0.075	0.116 #	0.170** #	− 0.043
	EMA Minutes	− 0.027 #	− 0.073	0.041 #	− 0.075	− 0.078
	Recall VRS	− 0.180** #	0.039	− 0.118	− 0.061	− 0.156*
	EMA VRS	− 0.234*** #	− 0.073	0.146 #	0.042 #	− 0.003
Physical activity	***Accelerometry***	***− 0.117***	***− 0.083***	0.225***	0.195***	***0.082***
	Recall Minutes	0.121 #	0.031	− 0.009 #	0.041 #	0.077
	EMA Minutes	0.234*** #	0.136* #	− 0.125* #	− 0.035 #	0.009
	Recall VRS	0.004	0.111 #	0.076 #	0.062 #	− 0.007
	EMA VRS	0.139* #	− 0.023	− 0.052 #	0.035 #	0.107

For individual correlations, * *p* < .05, ** *p* < .01, and *** *p* < .001.

# indicates that within the Sedentary Behavior and Physical Activity sections of the table, the marked correlation coefficient is significantly different than the correlation for accelerometry and the demographic variable shown in the row heading.

 The H3a hypothesis states that momentary measurements will have associations with the criterion variables that are more similar to the same associations with criterion variables and accelerometry compared with associations based on recall measurements. The H3b hypothesis states that quantitative scale associations will be more similar to accelerometry associations compared with associations with recall scales.

For age, accelerometry indicates significantly more sedentary behavior at older ages and a weaker, yet significant, negative association with PA. In marked contrast, 7 of 8 of the self-report associations with age have the opposite sign and were significantly different from the accelerometry associations. For gender, 3 of 4 self-report sedentary measures showed associations that were similar in magnitude to those for accelerometry, but recall VRS did not. However, for physical activity half of the self-reports were different from accelerometry. For relationships with education, 3 of 4 sedentary self-report measures were in the opposite direction and were significantly different than accelerometry. An inverse pattern was found for relationships with PA, where all four self-reports were in the opposite direction and different than accelerometry. For relationships with income, 2 of 4 SB self-reports were opposite and different than accelerometry, and, for PA all correlations with self-reports were significantly lower than the accelerometry correlation and there were no differences between accelerometry and self-reports for SB or PA for race.

Finally, the overall patterns observed in the correlation matrix including all demographic variables showed that neither was EMA clearly superior to recall reports (z = 1.43, *p* = .152), nor was self-reporting using a quantitative format superior to VRS responses (z = 0.56, *p* = .579) in terms of reproducing the associations of accelerometry with demographic variables.

## Discussion

Concerns about the validity of self-reports are well-known and improvements to existing techniques would be welcome. This study tested two techniques with the potential to improve self-report validity: reducing the length of the reporting interval (under 2 h versus 7-days) and using a response scale with non-relativistic options (quantitative duration versus verbal responses). A feature of the study design was that direct assessments of self-report validities were possible by contrasting self-reports with a reference standard for the outcome variables, accelerometry-recorded activity, allowing clear conclusions regarding the concurrent criterion validity and construct validity of self-reports.

We first consider concurrent criterion validity of the two techniques. Regarding mean level differences, both momentary and recall self-reporting resulted in estimates of minutes in sedentary behavior that were lower than those recorded by accelerometry. However, the momentary average was only 12% lower than accelerometry whereas the recall average was 30% lower. These findings are consistent with prior research, where individuals believed that they were less sedentary than they were by objective accounts^28^. Yet the error, especially for momentary self-reports, was not very large. At least for sedentary behavior, the momentary approach seems reasonable in terms of estimating the number of minutes of engagement. On the other hand, self-reporting of physically active minutes indicated many more minutes of activity for both recall and momentary approaches than were shown by accelerometry measures. Relative to accelerometry, recall was 155% higher and momentary was 380% higher. This finding is also consistent with prior studies^29^ and is in accord with the low number of sedentary minutes reported and with the view that people see themselves as physically healthier than objective measures indicate. Thus, self-reporting of PA for momentary and recall measurement yields duration estimates that are greatly inflated.

We speculate that the large level divergences in self-reports and accelerometry may, though, be attributable in the implicit definitions that participants used for PA versus the way the accelerometry algorithm calculated PA minutes. Regarding the participants, an attempt to standardize reporting of SB and PA was implemented in the study training protocol by providing definitions of the levels for physical activity; moreover, each assessment question included written examples of activities representing an activity level. Definitions and examples were roughly consistent with the categorization of physical activity used by the accelerometry algorithm. Despite these efforts to encourage participants to adopt the definitions, this may not have helped as evidenced by the large discrepancies between accelerometry and self-report (both momentary and recall) for PA. In this case the discrepancies may, at least in part, be viewed as a measurement artifact.

An overall takeaway from these results is that self-reporting is more accurate for SB than it is for PA and that EMA estimates were relatively good for SB. This was not the case for PA. Thus, we may conclude that this is partial support for EMA having criterion validity based on the analysis of mean levels.

A second method for determining concurrent criterion validity was by examination of associations that the reference standard had with self-reports. Two issues were addressed here – first, whether the momentary associations had stronger associations with accelerometry than recall and, second, whether quantitative measurement had stronger associations than verbal response scales. For sedentary behavior, aggregated momentary reports correlated substantially more strongly with accelerometry than did recall of sedentary minutes (0.62 versus 0.36). However, for physically active behaviors, there was no difference in the same correlations. We conclude, then, that there is partial support for the hypothesis that momentary assessment is more concurrently valid than recall, but the results for PA reduce our enthusiasm for this general conclusion.

Second, the were no differences in the concurrent validity for VRS versus quantitative duration scales. None of the correlations for SB or for PA revealed significant differences for the different scales’ associations with accelerometry, contrary to the hypothesized results. We discuss this finding in the section on construct validity below.

An issue remains about whether the observed validity coefficients are acceptable given the relatively small amount of variance shared among the measures, which is consistent with prior studies on recall questionnaire and accelerometry comparisons that showed even lower associations^11,30^. Given conventional validity thresholds of correlations of 0.7 or 0.8, even the higher momentary correlation may not be viewed as acceptable. The relatively small amount of explained variance in the majority of associations with accelerometry suggests considerable error in either or both self-reported measures and/or accelerometry-derived variables.

Our third hypothesis addressed an aspect of construct validity based on contrasting correlations of external variables with the reference standard and contrasting those findings with correlations of self-reports with the same external variables. Self-report associations for three of the five demographic criterion variables did not adequately reproduce the accelerometry associations. The differences were not simple diminutions of the associations of self-reports with demographics relative to the associations for accelerometry, which could have been the case if self-reports were less reliable than accelerometry. On the contrary, most of the associations for three demographic variables were in the opposite direction of the associations for accelerometer. For example, although accelerometry indicated that higher age was associated with *more* sedentary behavior, *all* types of self-reports indicated that higher age was associated with *less* sedentary behavior. As mentioned earlier, prior studies have often, but not always, reported findings consistent with these^11,30^, but these prior associations were for *recalled* self-reports (e.g., one-week) and accelerometry. To our knowledge, this is the first time that momentary assessments have shown comparable distortions. The upshot of these findings is that if the purpose of a research project was to examine aging and sedentariness and it only collected self-report data on activity, then the conclusions would be reversed compared to conclusions generated from accelerometry data.

Moreover, contrary to the hypothesized results was the observation that assessments using brief recall were not better (relative to the accelerometry associations) than those relying on 7-day retrospective recall. These findings challenge a core rationale for employing EMA and associated techniques – that bias and error are mitigated by using brief recall and are, therefore, more valid measures than their retrospective counterparts. It may be speculated that at least some of the psychological processes that distort retrospective reports are also present in reporting with very brief reporting periods, at least for physical activity behaviors. The quantitative duration scales for SB and PA, which asked for numeric responses, should not have induced the relativistic comparisons that verbal descriptor may produce^31^, reducing the possibility that this issue explains the distortion. Yet other potential sources of distortion such as different frames of reference^32^ wherein respondents construct their responses using various comparison standards, may distort both VRS and quantitative measures. This is counter to hope that momentary reports eliminate or reduce the problems with retrospection and, hence, are preferable in all cases to retrospective reports.

Also contrary to the hypothesized result we found no evidence that the quantitative measures were superior to the VRS measures. For associations with age, VRS measurements had slightly stronger negative associations than the duration measurements, though all self-reports with age were in the opposite direction than that for accelerometry. We were hopeful that the quantitative questions with brief reporting periods would be systematically superior to VRSs, but this was not the case.

There is an apparent contradiction between the direct associations between momentary measures and accelerometry tested in hypothesis 2 versus the construct validation results tested in hypothesis 3. The direct associations indicated weak to modest criterion validity. This could be interpreted as implying that self-reports are a reasonable substitute for accelerometry measures. Yet the construct validity analyses yielded a very different conclusion: self-reports were not reasonable substitutes for accelerometry measures for exploring associations with some external variables. How could the discordant construct associations reported here between accelerometry and self-reports with demographic outcomes be reconciled with positive concurrent validity? The sizable unexplained variance between accelerometry and self-reports in the direct associations may suggest an answer. It must be that the portions of unexplained variance in self-report and accelerometry associations have differential associations with age, education, and income, and that those unique associations allowed the observed inversions in associations. Thus, the nonoverlapping variance in accelerometry has distinctive associations with external variables whereas the unshared variance in the self-reports has different (and sometimes, opposite) relationships. We think that additional examination of this speculation could lead to insights into the cognitive processes underpinning the construction of self-reports. Notably, insights such as these are not possible without a reference standard for comparison.

Our overall conclusions about self-report validity are formally restricted to self-reporting of sedentary behavior and physical activity. In other words, we cannot argue for the generalizability of the findings to other types of self-reporting, such as the purely subjective phenomena mentioned in the background material. They are also limited to the between-person associations examined in this study. We have presented no evidence regarding the momentary techniques pertinent to within-person associations. Therefore, we do not currently consider these results a strong challenge to the criterion or construct validity of self-reports. That said, the findings will hopefully stimulate future work with outcomes that allow a direct comparison of momentary findings with appropriate reference standards. We emphasize that this research is paramount given the current belief that momentary data capture techniques, including EMA and ESM, are “gold standards” for measuring subjective states and that quantitative scales are superior to verbal response scales. This was not convincingly the case within the context of physical activity measures. Although the “gold standard” belief may be true, our results intimate that the statement be viewed with healthy skepticism.

## Data Availability

The datasets used and/or analyzed during the current study available from the corresponding author on reasonable request.

## References

[1] Broderick, J. E., Stone, A. A., Calvanese, P., Schwartz, J. E. & Turk, D. C. Recalled pain ratings: A complex and poorly defined task. *J. Pain*. **7**, 142–149 (2006). 16459280 10.1016/j.jpain.2005.09.012

[2] Conner, T. S. & Barrett, L. F. Trends in ambulatory self-report: the role of momentary experience in psychosomatic medicine. *Psychosom. Med.* **74**, 327–337 (2012). 22582330 10.1097/PSY.0b013e3182546f18 PMC3372543

[3] Norman, G. R., Stratford, P. & Regehr, G. Methodological problems in retrospective ocmputation of responsiveness to change: the lessons of Cronbach. *J. Clin. Epidemiol.* **50**, 869–879 (1997). 9291871 10.1016/s0895-4356(97)00097-8

[4] Bolger, N., Davis, A. & Rafaeli, E. Diary methods: capturing life as it is lived. *Annu. Rev. Psychol.* **54**, 579–616 (2003). 12499517 10.1146/annurev.psych.54.101601.145030

[5] Shiffman, S., Stone, A. A. & Hufford, M. R. Ecological momentary assessment. *Annu. Rev. Clin. Psychol.* **4**, 1–32 (2008). 18509902 10.1146/annurev.clinpsy.3.022806.091415

[6] Troiano, R. P. et al. Physical activity in the united States measured by accelerometer. *Med. Sci. Sports Exerc.* **40**, 181–188 (2008). 18091006 10.1249/mss.0b013e31815a51b3

[7] Freedson, P. S. & Miller, K. Objective monitoring of physical activity using motion sensors and heart rate. *Res. Q. Exerc. Sport*. **71** (Suppl 2), 21–29 (2000). 25680009 10.1080/02701367.2000.11082782

[8] Doherty, A. et al. Large scale population assessment of physical activity using wrist worn accelerometers: the UK biobank study. *PLoS One*. **12**, e0169649 (2017). 28146576 10.1371/journal.pone.0169649 PMC5287488

[9] Sylvia, L. G., Bernstein, E. E., Hubbard, J. L., Keating, L. & Anderson, E. J. Practical guide to measuring physical activity. *J. Acad. Nutr. Diet.* **114**, 199–208 (2014). 24290836 10.1016/j.jand.2013.09.018 PMC3915355

[10] Doma, K., Speyer, R., Leicht, A. S. & Cordier, R. Comparison of psychometric properties between usual-week and past-week self-reported physical activity questionnaires: a systematic review. *Int. J. Behav. Nutr. Phys. Act.* **14**, 10 (2017). 28137268 10.1186/s12966-017-0470-6 PMC5282723

[11] Dyrstad, S. M., Hansen, B. H., Holme, I. M. & Anderssen, S. A. Comparison of self-reported versus accelerometer-measured physical activity. *Med. Sci. Sports Exerc.* **46**, 99–106 (2014). 23793232 10.1249/MSS.0b013e3182a0595f

[12] Csikszentmihalyi, M. & Hunter, J. Happiness in everyday life: the uses of experience sampling. *J. Happiness Stud.* **4**, 185–199 (2003).

[13] Stone, A. A. & Shiffman, S. Ecological momentary assessment (EMA) in behavioral medicine. *Ann. Behav. Med.* **16**, 199–202 (1994).

[14] Schwarz, N. Self-reports. How the questions shape the answers. *Am. Psychol.* **54**, 93–105 (1999).

[15] Walentynowicz, M., Schneider, S., Junghaenel, D. U. & Stone, A. A. Vague quantifiers demonstrate little susceptibility to frame of reference effects. *Appl. Res. Qual. Life*. **17**, 317–331 (2022). 35330704 10.1007/s11482-020-09889-0 PMC8939886

[16] Schneider, S. & Stone, A. A. The meaning of vaguely quantified frequency response options on a quality of life scale depends on respondents’ medical status and age. *Qual. Life Res.* **25**, 2511–2521 (2016). 27071685 10.1007/s11136-016-1293-7 PMC5345903

[17] Song, J., Howe, E., Oltmanns, J. R. & Fisher, A. J. Examining the concurrent and predictive validity of single items in ecological momentary assessments. *Assessment* **30**, 1662–1671 (2023). 36004406 10.1177/10731911221113563 PMC10248304

[18] Cronbach, L. J. & Meehl, P. E. Construct validity in psychological tests. *Psychol. Bull.* **52**, 281–302 (1955). 13245896 10.1037/h0040957

[19] Millsap, R. E. Measurement invariance, predictive invariance, and the duality paradox. *Multivar. Behav. Res.* **30**, 577–605 (1995). 10.1207/s15327906mbr3004_6 26790049

[20] Weber, A. et al. Large-scale assessment of physical activity in a population using high-resolution hip-worn accelerometry: the German National cohort (NAKO). *Sci. Rep.* **14**, 7927 (2024). 38575636 10.1038/s41598-024-58461-5 PMC10995156

[21] Ekblom-Bak, E. et al. Accelerometer derived physical activity patterns in 27.890 middle-aged adults: the SCAPIS cohort study. *Scand. J. Med. Sci. Sports*. **32**, 866–880 (2022). 35080270 10.1111/sms.14131 PMC9302631

[22] Kapteyn, A., Angrisani, M., Darling, J. & Gutsche, T. The Understanding America study (UAS). *BMJ Open.* **14**, e088183 (2024). 39448221 10.1136/bmjopen-2024-088183 PMC11499792

[23] Bassett, D. R. et al. Detection of lying down, sitting, standing, and stepping using two activpal monitors. *Med. Sci. Sports. Exerc.* **46**, 2025–2029 (2014). 24598698 10.1249/MSS.0000000000000326

[24] Lyden, K., Keadle, S. K., Staudenmayer, J. & Freedson, P. S. The activpal™ accurately classifies activity intensity categories in healthy adults. *Med. Sci. Sports. Exerc.* **49**, 1022–1028 (2017). 28410327 10.1249/MSS.0000000000001177 PMC5469371

[25] Thurman, S. M. et al. Individual differences in compliance and agreement for sleep logs and wrist actigraphy: A longitudinal study of naturalistic sleep in healthy adults. *PLoS One*. **13**, e0191883 (2018). 29377925 10.1371/journal.pone.0191883 PMC5788380

[26] Tudor-Locke, C., & Rowe, D.A. Using cadence to study free-living ambulatory behaviour. *Sports Med.* **42**(5), 381–398. PMID: 22462794 (2012). 10.2165/11599170-000000000-00000. 10.2165/11599170-000000000-00000 22462794

[27] Steiger, J. H. Tests for comparing elements of a correlation matrix. *Psycjological Bull.* **87**, 245–251 (1980).

[28] Prince, S. A. et al. A comparison of self-reported and device measured sedentary behaviour in adults: a systematic review and meta-analysis. *Int. J. Behav. Nutr. Phys. Act.* **17**, 31 (2020). 32131845 10.1186/s12966-020-00938-3 PMC7055033

[29] Prince, S. A. et al. A comparison of direct versus self-report measures for assessing physical activity in adults: a systematic review. *Int. J. Behav. Nutr. Phys. Act.* **5**, 56 (2008). 18990237 10.1186/1479-5868-5-56 PMC2588639

[30] Lee, P. H. et al. Performance of the international physical activity questionnaire (short form) in subgroups of the Hong Kong Chinese population. *Int. J. Behav. Nutr. Phys. Act.* **8**, 81 (2011). 21801461 10.1186/1479-5868-8-81 PMC3157408

[31] Tourangeau, R. in *In Cognitive Aspects of Survey Methodology: Building a Bridge between Disciplines*. 73–100 (eds Jabine, T., Straf, M., Tanur, J. & Tourangeau, R.) (National Academy, 1984).

[32] Fienberg, S. E., Loftus, E. F. & Tanur, J. M. Cognitive aspects of health survey methodology: an overview. *Milbank Mem. Fund Q. Health Soc.* **63**, 547–564 (1985). 3852078

